# Challenging Assumptions about Lead and IQ: Effects Increase, Not Decrease, in Older Children

**Published:** 2005-05

**Authors:** Bob Weinhold

The concentration of lead in children’s blood peaks at about age 2 years and then declines as hand-to-mouth activity tends to drop off. Much of the practice and research concerning lead poisoning is based on the belief that the most damage is done by that peak. However, lead’s effects on IQ cannot be detected until about 4 or 5 years of age, when IQ becomes testable. Thus, researchers assume, if we wish to know the lowest level at which lead causes damage, we have to measure blood lead in 2-year-olds and follow them, and if we wish to prevent lead toxicity from occurring, we should focus on 2-year-olds. Both assumptions, and the outcomes they encourage, may be incorrect, concludes a U.S. research team after analyzing data from a study that began in 1994 **[*EHP*** **113:597–601]**.

The Treatment of Lead-Exposed Children study was initially designed to evaluate whether a drug called succimer, which lowers blood lead, would reduce or prevent the effects of lead on IQ. The 780 participating children, selected in approximately equal numbers from clinical centers in Baltimore, Cincinnati, Newark, and Philadelphia, were regularly tested for blood lead concentration and given IQ tests from about age 2 years to about age 7.5 years. About half the children had taken succimer, while the others had taken a placebo. The drug lowered the children’s blood lead concentrations, but the group given succimer did no better on IQ tests than the group given placebo.

In the current study, researchers used the earlier data to evaluate the strength of the association between IQ and blood lead at various ages, and whether blood lead at age 2 years affected IQ at ages 5 and 7 more than blood lead measured at the older ages. The team examined blood lead and intelligence data from ages 2, 5, and 7, as well as many other factors, such as race, sex, language spoken, caregiver’s IQ, and parent’s education, employment, and status as a single parent.

Contrary to most current thinking, which assumes that blood lead concentration at age 2 is the best predictor of IQ at ages 5 and 7, this team found that concurrent blood lead concentration had the strongest association with IQ, and the older the child, the stronger the association. This was true even though blood lead concentrations dropped progressively as the children aged. A few other studies had found somewhat similar results, but the researchers say the size of this study and the quality of its data reinforce the strength of the findings.

This study does have some drawbacks. For instance, the investigators had no data reflecting how much caregivers interacted with and stimulated the children, which can influence a child’s intelligence. In addition, the children selected for the original study weren’t representative of the population as a whole. For instance, their initial blood lead concentrations were higher than those of most U.S. children today, 77% of the children were black, 97% were receiving public assistance, and 72% lived in single-parent households.

Nonetheless, the team concludes that ongoing lead contamination has a significant effect on a child’s intelligence, emphasizing the importance of testing for and reducing lead contamination in the environment of children much older than typically targeted. They also say the findings, if they hold up and are accepted, would relax an important limitation on lead studies, allowing future efforts to include subjects who were not tested for lead at age 2.

